# From Crystal Structures of RgIA4 in Complex with *Ac*-AChBP to Molecular Determinants of Its High Potency of α9α10 nAChR

**DOI:** 10.3390/md19120709

**Published:** 2021-12-17

**Authors:** Si Pan, Yingxu Fan, Xiaopeng Zhu, Yi Xue, Sulan Luo, Xinquan Wang

**Affiliations:** 1 Key Laboratory of Translational Tumor Medicine in Fujian Province, Putian University, Putian 351100, China; plar000@163.com; 2 Tsinghua-Peking Joint Center for Life Sciences, School of Life Sciences, Tsinghua University, Beijing 100084, China; fanyx17@mails.tsinghua.edu.cn; 3 Medical School, Guangxi University, Nanning 530004, China; biozxp@163.com; 4 Key Laboratory of Tropical Biological Resources of Ministry of Education, Key Laboratory for Marine Drugs of Haikou, School of Life and Pharmaceutical Sciences, Hainan University, Haikou 570228, China; 5 Beijing Advanced Innovation Center for Structural Biology, Collaborative Innovation Center for Biotherapy, The Ministry of Education Key Laboratory of Protein Science, School of Life Sciences, Tsinghua University, Beijing 100084, China

**Keywords:** acetylcholine binding protein, nicotinic acetylcholine receptors, α-conotoxin, RgIA, RgIA4, crystal structure, molecular dynamics simulation

## Abstract

α9-containing nicotinic acetylcholine receptors (nAChRs) have been shown to play critical roles in neuropathic pain. The α-conotoxin (α-CTx) RgIA and its analog RgIA4 were identified as the most selective inhibitor of α9α10 nAChR. However, the mechanism of their selectivity toward α9α10 nAChR remains elusive. Here, we reported the co-crystal structure of RgIA and RgIA4 in complex with *Aplysia californica* acetylcholine binding protein (*Ac*-AChBP) at resolution of 2.6 Å, respectively. Based on the structure of the complexes, together with molecular dynamic simulation (MD-simulation), we suggested the key residues of α9α10 nAChR in determining its high affinity for RgIA/RgIA4. This is the first time the complex between pain-related conotoxins and *Ac*-AChBP was reported and the complementary side of α9 subunit in binding of the antagonists shown. These results provide realistic template for the design of new therapeutic in neuropathic pain.

## 1. Introduction

Nicotinic acetylcholine receptors (nAChRs) are the best characterized members of the pentameric ligand-gated ion channels (pLGICs) family that also includes receptors for serotonin (5-HT3), GABA_A_, Zinc-activated channels (ZAC receptor), and glycine receptors [1,2]. They can be further divided into two types: the neuronal type nAChRs and muscle-type nAChRs. Neuronal nAChRs are mainly located in the central and peripheral nervous systems and mediate fast neurotransmission [3]. In vertebrates, neuronal nAChRs are composed of a combination of eight α (α2-α7, α9-α10) and three β (β2-β4) subunits, forming either homopentamers or heteropentamers (e.g., α7, α4β2, α3β2, and α9α10 nAChRs) [4]. All α and β subunits share a similar architecture with an N-terminal extracellular ligand-binding domain, a transmembrane domain consisting of four α-helixes, and a short C-terminal extracellular tail. The binding of neurotransmitters acetylcholine to the extracellular domain induces structural rearrangements of the transmembrane domain, leading to a rapid opening of the central ion-conducting pore in the pentameric nAChRs [5].

Neuronal nAChRs are implicated in nicotine addiction, abuse of drugs, and various neurological and non-neurological diseases such as neuropathic pain, Alzheimer’s, Parkinson’s diseases, and inflammation [6]. Therefore, it is highly desirable to develop nAChR antagonists with high potency and selectivity as therapeutic agents and pharmacological tools. α-conotoxins (α-CTxs) from *Conus* genus are a family of small, disulfide-linked peptide neurotoxins that are competitive antagonists of nAChRs with unparalleled potency and selectivity [7]. Classical α-conotoxins are generally 12–20 amino acids in length with four cysteine residues in a framework of CC-C-C. Typically, α-conotoxin globular isomer with CysI-CysIII and CysII-CysIV connectivity and α-helical backbone is the naturally occurring bioactive form. Several α-conotoxins showing selectivity for different nAChR subtypes have been identified as potent antagonists of chronic pain, such as Vc1.1 and RgIA. Early publications suggested that Vc1.1 could selectively inhibit rat α9α10 nAChR with the affinity of 19 nM. Unfortunately, a clinical trial of Vc1.1 was dropped during phase IIa since its potency at human nAChR was 100-fold lower than that at rat nAChR [8]. Subsequently, Vc1.1 was reported to inhibit human dorsal root ganglion neuron via activating the GABA_B_ receptor [9]. Up to now, it is still unclear whether the inhibition of α9α10 nAChR or activation of GABA_B_ receptor is the analgesic mechanism. Our present study focused on α-CTx RgIA and its targeting receptor α9α10 nAChR. It was found that α9-containing nAChRs including α9α10 play critical roles in neuropathic pain after chemotherapy-induced neuropathy and traumatic injury to nerves [10]. The α9 and α10 subunits share a high sequence similarity, especially in the extracellular domain (ECD) region with ~77% identity [11]. In contrast to the α9 subunit, α10 subunits do not form functional receptors by themselves but do assemble with α9 subunits to form α9α10 heteropentamer [12]. RgIA, a 13-amino-acid α-conotoxin isolated from the carnivorous marine snail, is a highly selective α9α10 nAChR antagonist, showing >1000-fold higher potency in inhibiting α9α10 than other nAChRs. RgIA blocks rodent α9α10 nAChR with high potency and has been shown to be effective in rodent models of neuropathic pain [13]. However, it is approximately 300-fold less active on human α9α10 nAChR due to a single-site amino acid substitution in human α9 subunit, which limits potential therapeutic application of RgIA [14]. RgIA4, an engineered analog, was reported to exhibit high potency on both human and rodent α9α10 nAChR, and has been shown to effectively prevent oxliplatin-induced pain in rats and mice [15,16]. Therefore, the RgIA4/α9α10 pair is a critical and promising target for developing and optimizing therapeutic agents for prevention of chronic neuropathic pain.

Structural information of the ligand-binding site in the pentameric α9α10 receptor and its complex with RgIA would greatly advance our understanding of the molecular basis for the high potency of RgIA against the α9α10 nAChR. The α-CTx-binding site in the pentameric nAChR usually consists of the principal (+) side of the ECD of an α subunit and the complementary (−) side of the ECD of the adjacent α or β subunit. Recently, the crystal structure of monomeric extracellular domain of α9 subunit bound with RgIA was determined [17]. However, this structure only displayed the principal side between α9-ECD and RgIA, and the complementary side of α10-ECD and the interaction with RgIA is still unknown. Therefore, we have not fully understood how RgIA achieved high inhibitory activity against the rodent α9α10 receptor, as well as how the modification in RgIA4 closes the affinity gap across the rodent and human receptors.

Currently, a few pentameric nAChR receptor structures including Torpedo nAChR, α7 nAChR chimera, α4β2 nAChR, and human α2 ECD pentamer have been determined by X-ray diffraction and cyro-EM methods, possibly due to the difficulties encountered in expressing and assembling the pentameric receptors [18,19,20,21]. One significant progress in the structural and functional studies of nAChRs was the discovery and crystallization of acetylcholine-binding protein (AChBP), which is a soluble homopentamer and firstly isolated from the snail *Lymnaea stagnalis* [22,23]. Sequence alignment showed that AChBPs are most closely related to the ECDs of nAChRs, especially the α-subunits in nAChRs, and nearly all conserved residues in nAChRs for ligand binding are present in AChBP [22]. Thus, pentameric AChBP is an excellent structural and functional homolog of the ECDs of most nAChRs. The first isolated and crystalized AChBP was from *Lymnaea stagnalis* (*Ls*-AChBP), but most crystal structures of different α-CTxs have been solved for the complexes with the AChBP from *Aplysia californica* (*Ac*-AChBP) and served as important models for studying α-CTx/nAChR interactions at the atomic level.

Here, we reported the co-crystal structures of *Ac*-AChBP bound with RgIA and RgIA4, respectively. We analyzed the structural interfaces in these two complexes and revealed detailed interactions of RgIA and RgIA4 with *Ac*-AChBP, respectively. Based on the determined complex structures of α-CTxs with *Ac*-AChBP, we also built the complexes of α9α10 nAChR with RgIA and RgIA4 by homologous modeling and MD-simulation. By analyzing these two α-CTx/α9α10 complexes, we also revealed the molecular basis underlying how the modifications of two residues in RgIA4 result in additional interactions and increase the affinity with human α9α10 nAChR. Since the α9α10 nAChR is an important pharmacotherapeutic target for the pain model, our study provides significant implications for the design of highly selective therapeutic α-conotoxin analogs for use against nAChR-related diseases.

## 2. Results

### 2.1. Overall Structure of RgIA and RgIA4 Bound to Ac-AChBP

**Figure 1:**
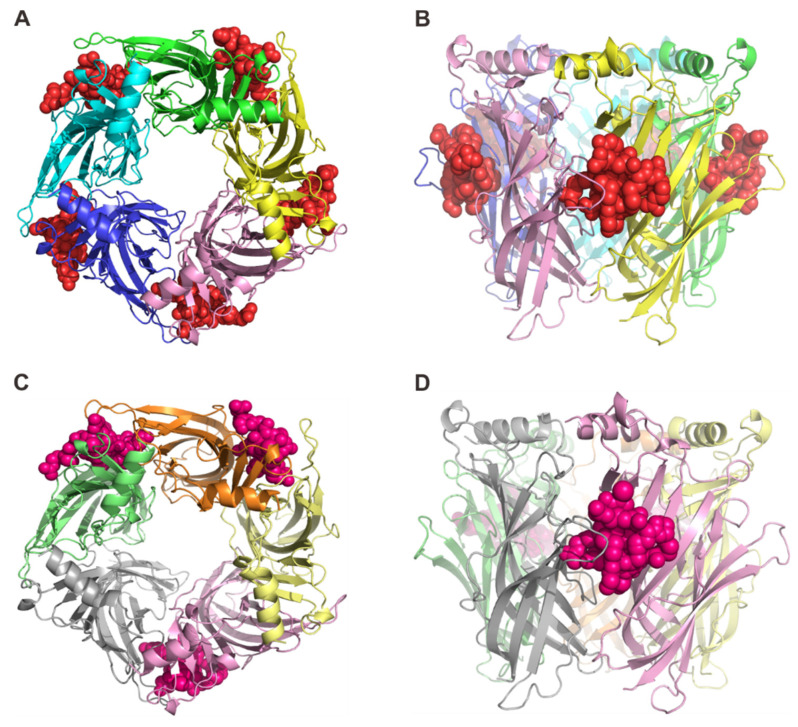
The X-ray crystal structure of *Ac*-AChBP in complex with RgIA and RgIA4, respectively. The PDB IDs of these complexes are 7EGR (RgIA-*Ac*-AChBP) and 7EGX (RgIA4-*Ac*-AChBP). (**A**) The top view of the pentameric structure with five *Ac*-AChBP protomers, each in different colors and five α-CTx RgIA molecules in red. (**B**) The side view of the pentamer with RgIA molecules (in red). (**C**) The top view of the pentameric structure with five *Ac*-AChBP protomers, each in different colors and with RgIA analog RgIA4 molecules in magenta. (**D**) The side view of the pentamer with RgIA molecules (in magenta).

**Figure 2:**
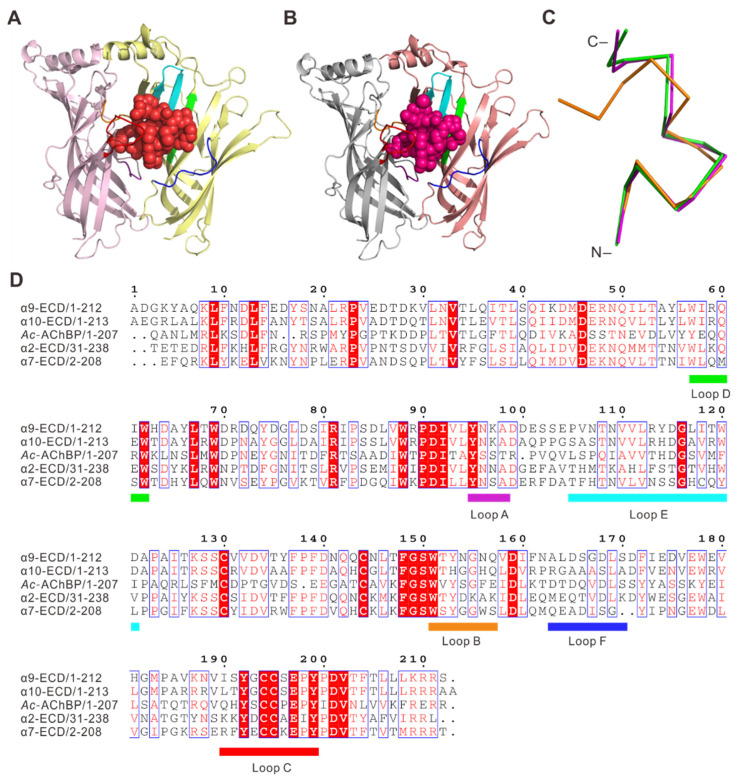
Schematic diagram of *Ac*-AChBP with RgIA/RgIA4 and homologous proteins. (**A**) The side view of two adjacent AChBP protomers of the pentamer with a bound RgIA molecule (in red). (**B**) The side view of two adjacent AChBP protomers of the pentamer with a bound RgIA4 molecule in magenta. (**C**) Superposition of RgIA/RgIA4 in different state. AChBP-bound RgIA in magenta, AChBP-bound RgIA4 in green, NMR structure of free-state RgIA in orange. Bottom, Multiple sequence alignment of RgIA, RgIA4, GIC, and LvIA. Disulfide bridges are shown in Cys1-Cya3 and Cys2-Cys4. (**D**) Sequence alignment of *Ac*-AChBP, human α9, α10, α2, and α7 nAChR ECD.

To investigate the binding at atomic details, we determined the complex structures of *Ac*-AChBP bound with RgIA and RgIA4, respectively. The amino acid sequence of RgIA is GCCSDPRCRYRCR-, and the folded peptide has two disulfide bonds connecting Cys2 to Cys8 and Cys3 to Cys12. The RgIA4 was derived from RgIA by substituting Arg9 and Tyr10 with Citrulline (represented as CIR) and 3-Iodo-Tyrosine (represented as IYR), respectively. The RP-HPLC and electrospray ionization mass spectrometry (ESI-MS) were utilized to purify and characterize the synthesized and refolded RgIA and RgIA4 (Figure S1). The *Ac*-AChBP was expressed in Hi5 insect cells and purified by gel-filtration column. Both complex structures, RgIA/*Ac*-AChBP and RgIA4/*Ac*-AChBP, were determined by X-ray crystallography at 2.6 Å resolution (Figure 1, Table S1). In these structures, five monomers of *Ac*-AChBP assemble into a homopentamer, resembling a windmill toy with petal-like monomers. The overall pentameric structure is analogous to the quaternary structures of nAChR ECDs (Figure 1). As shown in the Figure 1, the *Ac*-AChBP homopentamer has five ligand binding sites, each located in a cleft between two adjacent subunits (Figure 1B,D). All five ligand-binging sites are occupied by the RgIA in the RgIA/*Ac*-AChBP complex (Figure 1A), and only three RgIA4 peptides were found in the RgIA4/*Ac*-AChBP complex (Figure 1C). The loops A, B, and C of one *Ac*-AChBP monomer are involved on the ligand binding at the principal side and the loops D, E, and F and several β-strands of another monomer are involved in the ligand binding at the complementary side (Figure 2D). In the ligand-binding site, the RgIA and RgIA4 overall structures were very similar with a central short helix and unstructured N- and C-terminus (Figure 2A,B). Structural comparison showed that the RgIA in the complex and unbound RgIA have obvious backbone conformational differences, indicating the binding with *Ac*-AChBP could probably induce conformational changes in RgIA (Figure 2C).

### 2.2. Interactions of RgIA with Ac-AChBP

**Figure 3:**
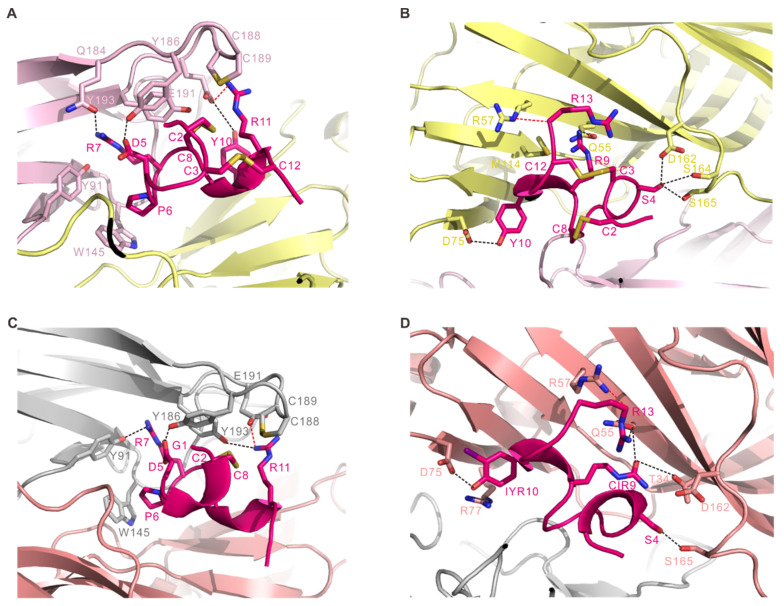
*Ac*-AChBP-α-CTx binding interfaces. (**A**) The interactions on the principal side of RgIA-*Ac*-AChBP complex. Residues Asp5, Arg7, and Tyr10 of the RgIA form hydrogen bonds (represented by black dashed line) with Tyr186, Gln184, and Glu191 of *Ac*-AChBP, respectively. Salt bridge (represented by the red dashed line) is also observed between Arg11 of RgIA and Glu191 of *Ac*-AChBP. (**B**) The complementary side of the RgIA-*Ac*-AChBP complex. Hydrogen bonds are involved in Residues Ser4, Arg9, and Tyr10 of RgIA and Gln55, Asp75, Asp162, Ser164, and Ser165 of *Ac*-AChBP. Residue Arg13 of RgIA forms a salt bridge with Arg57 of *Ac*-AChBP. (**C**) The principal side of RgIA4-*Ac*-AChBP complex. Residues Asp5, Arg7, and Arg11 of the RgIA4 form hydrogen bonds with Tyr186, Tyr91, and Tyr193 of *Ac*-AChBP, respectively. Residue Arg11 of RgIA4 forms a salt bridge with Glu191 of *Ac*-AChBP. (**D**) The complementary side of RgIA4-*Ac*-AChBP complex. Hydrogen bonds are involved in Residues Ser4, CIR9, IYR10, and Arg13 of RgIA4 and Thr34, Gln55, Asp75, and Ser165 of *Ac*-AChBP. Residue Arg13 of RgIA4 forms a salt bridge with Arg57 of *Ac*-AChBP.

To fully reveal the interactions between RgIA and *Ac*-AChBP, we performed a structural analysis of the principal and complementary sides in the RgIA/*Ac*-AChBP complex (Figure 3A and Table 1). A mixture of hydrophobic and hydrophilic interactions occurred on the principal interaction side, and the *Ac*-AChBP-contacting residues were mainly from the loop B (Trp145) and loop C (Gln184-Tyr193) (Figure 3A and Table 1). Like other structures of typical α-CTxs bound to *Ac*-AChBP, the Cys2-Cys8 disulfide bridge of the RgIA stacked onto the vicinal Cys188-Cys189 disulfide bond of the *Ac*-AChBP. Hydrogen bonding interactions were formed between Asp5, Arg7, and Tyr10 of RgIA and Gln184, Tyr186, and Glu191 from loop C of the *Ac*-AChBP. Additionally, Glu191 of *Ac*-AChBP formed a salt bridge interaction with Arg11 of RgIA. Other interactions included the contacts of Pro6 and Arg7 of RgIA with Trp145 (loop B) and Tyr91 (loop A) of *Ac*-AChBP, respectively. The complementary interaction side was formed by β-strands, loop D (Gln55, ArG57), loop E (Met114), and loop F (Asp162, Ser164, and Ser165) of adjacent *Ac*-AChBP (Figure 3B and Table 1). On this side, a significant interacting residue of RgIA was Ser4, whose side chain resided in a pocket contacting with Asp162 (loop F), Ser164 (loop F), and Ser165 of *Ac*-AChBP through several hydrogen bonds. Another hydrogen bond was formed between Asp75 of *Ac*-AChBP and Tyr10 of RgIA. Besides, a salt bridge interaction was formed between Arg57 (loop D) of *Ac*-AChBP and Arg13 of RgIA.

### 2.3. Interactions of RgIA4 with Ac-AChBP

Unlike RgIA showing ~300-fold higher potency on the rodent α9α10 nAChR, the analog RgIA4 with Arg9 and Tyr10 replaced by CIR and IYR closed the affinity gap across the rodent and human α9α10 nAChR. To provide insights into the underlying molecular basis, we also determined the complex structure of RgIA4 with *Ac*-AChBP. Figure 3C,D show the structural features of the principal and complementary sides in the complex, respectively. On the principal side, *Ac*-AChBP residues Tyr91, Trp145, Tyr186, Cys188, Cys189, Glu191, and Tyr193 play key roles in binding RgIA4. Similar to other conotoxin/*Ac*-AChBP complexes, the Cys2-Cys8 disulfide bridge of the RgIA4 also stacked onto the vicinal Cys188-Cys189 disulfide bond of the *Ac*-AChBP. Different from RgIA/*Ac*-AChBP complex, the hydrogen bonding interactions involved Tyr193 (loop C) and Tyr91 (loop A) of the *Ac*-AChBP and Arg7 and Arg11 of RgIA4, respectively. Like the RgIA/*Ac*-AChBP complex, additional hydrophilic interactions such as the salt bridge also formed between Arg11 of RgIA4 and Glu191 (loop C) (Figure 3C and Table 1). On the complementary side, salt bridge interaction was also formed between Arg13 of RgIA4 and Arg57 (loop D) of the *Ac*-AChBP-like RgIA/*Ac*-AChBP complex. Residues CIR9 and IYR10 of the RgIA4 were involved in the interactions on the complementary side, where the contacting *Ac*-AChBP residues were mainly from the β-strands and loop D (Figure 3D). Different from RgIA/*Ac*-AChBP complex, on this side, hydrogen bonds included Ser4 (RgIA4) to Ser165 (*Ac*-AChBP), CIR9 (RgIA4) to Thr34 and Gln55 (*Ac*-AChBP), IYR10 (RgIA4) to Asp75 (*Ac*-AChBP), and Arg13 (RgIA4) to Gln55 (loop D of *Ac*-AChBP) (Figure 3D and Table 1).

### 2.4. Homology Modelling of Human α9α10 nAChR and Docking with RgIA and Its Mutant

**Figure 4:**
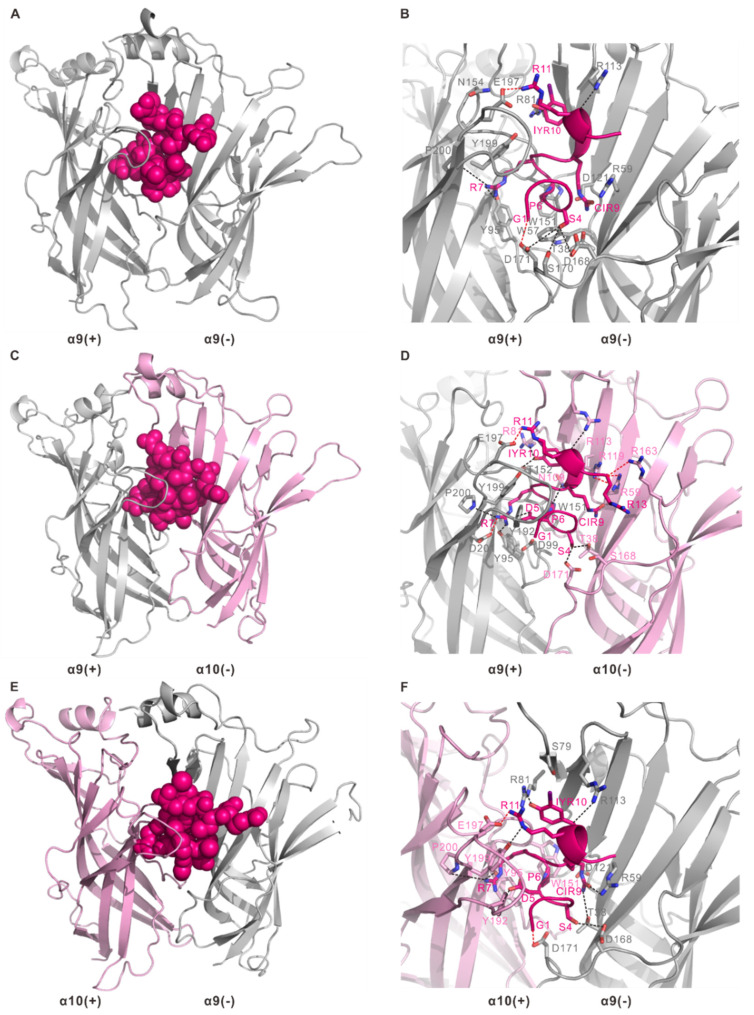
Molecular dynamics models of human α9α10 nAChR bound to RgIA4. (**A**,**B**) RgIA4 bound to the α9(+)/α9(−) interface. (**C**,**D**) RgIA4 bound to the α9(+)/α10(−) interface. (**E**,**F**) RgIA4 bound to the α10(+)/α9(−) interface. Hydrogen bonds and salt bridges are represented by black dashed lines and red dashed lines, respectively.

To gain further insight into the interactions of RgIA and RgIA4 with human α9α10 nAChR, especially the increasing binding of RgIA4, we built the ECD pentamers of human α9α10 nAChR with two possible stoichiometries (α9)_2_(α10)_3_ and (α9)_3_(α10)_2_ bound with RgIA and RgIA4 by homologous modeling, followed by MD-simulations. The models were constructed based on the X-ray crystal structures of the RgIA/*Ac*-AChBP and RgIA4/*Ac*-AChBP complexes. Subsequently, the generated models were validated in terms of stereochemical quality by Ramachandran plot (Figure S2). The results revealed that in over 90% of residues, two models were in the most favored and additional allowed regions, reflecting the good-quality models predicted and worth investigating further. There are three possible ligand binding sites in two different α9α10 nAChR pentamers: α9(+)/α10(−), α9(+)/α9(−), and α10(+)/α9(−). Considering that interactions of RgIA with human α9α10 nAChR have been modeled and described in previous studies, here we focused on the interaction of RgIA4 with receptor at the α9(+)/α10(−), α9(+)/α9(−), and α10(+)/α9(−) interface (Figure 4). These results about the interactions of RgIA with human α9α10 nAChR are shown in Figure S3.

At the α9(+)/α9(−) binding interface (Figure 4A,B), two α9 subunits served on the principal side and complementary side, respectively. As shown in Figure 4B, residues Tyr95, Trp151, Asn154, Glu197, Tyr199, and Pro200 of the α9 subunit are the key contacting residues on the principal side. Specific interactions included Tyr95 and Pro200 forming a hydrogen bond with Arg7 of RgIA4 and Glu197 interacts with Arg11 of RgIA4 through a salt bridge. On the complementary side, a notable interacting residue in the RgIA4 was Ser4, whose side chain resided in a pocket contacting with Thr38, Asp168, Ser170, and Asp171 of the other α9 subunit. Hydrogen bonds were formed between Ser4 of RgIA4 with Asp168, Ser170, and Asp171 of the α9 subunit, respectively. Near this interacting pocket, a salt bridge was formed between Gly1 of RgIA4 and Asp171 of the α9 subunit. Additionally, other hydrogen bonding interactions occurred between IYR10 of RgIA4 and Arg81 and Arg113 of the α9 subunit, respectively. Residues Thr38, Trp57, Arg59, and Asp121 of the α9 subunit had van der Waals contacts with CIR9 of RgIA4.

At the α9(+)/α10(−) binding site (Figure 4C,D), the principal side exhibited subtle differences from that at the α9(+)/α9(−) interface, although the contacting partners were the same between the α9 subunit and RgIA4. Two significant exceptions were Thr152 and Asp201 of the α9 subunit, which were not involved in the interaction at the principal side of α9(+)/α9(−) binding interface. Thr152 and Asp201 of the α9 subunit interacted with IYR10 and Arg7 of RgIA4 through a hydrogen bond and salt bridge, respectively. Another exception is the hydrogen bond between Tyr192 of α9 subunit and Asp5 of RgIA4. On the complementary side, most interactions were similar to those of α9(+)/α9(−) binding interface. It is obvious to show that there were two binding pockets around Ser4 and Arg13 of RgIA4. The side chain of Ser4 was located in the pocket which is composed of Thr38, Ser168, and Asp171 of the α10 subunit. Additionally, Ser4 of RgIA4 formed hydrogen bonds with Thr38 and Asp171, respectively. Arg13 of RgIA4 interacted with Arg59, Arg119 and Arg163 through salt bridges. Compared with the α9(+)/α9(−) binding interface, there was an additional hydrogen bond between Arg81 and Arg113 of the α10 subunit and IYR10 of RgIA4 (Figure 4D).

At the α10(+)/α9(−) binding interface (Figure 4E,F), interactions were very similar to the α9(+)/α9(−) interface both on the principal side and complementary side. Like the α9(+)/α9(−) binding interface, Arg7 of RgIA4 interacted with Tyr95 and Pro200 of α10 subunit through hydrogen bonds. Additionally, Tyr199 of the α10 subunit interacted with Arg11 of RgIA4 through hydrogen, which was not involved in other interfaces at the principal side. On the complementary side, IYR10 of rgIA4 also formed hydrogen bonds with Arg81 and Arg113 of the α9 subunit. Compared with the other two interfaces, additional hydrogen bonds occurred between Thr38 and Arg59 of the α9 subunit and CIR9 of RgIA4. This difference may explain the closed affinity gap of RgIA4 across the rodent and human α9α10 nAChR.

Additionally, Tyr199 of α10 subunit interacted with Arg11 of RgIA4 through hydrogen, which was not involved in other interfaces at the principal side. On the complementary side, IYR10 of rgIA4 also formed hydrogen bonds with Arg81 and Arg113 of α9 subunit. Compared with the other two interfaces, additional hydrogen bonds occurred between Thr38 and Arg59 of α9 subunit and CIR9 of RgIA4. This difference may explain the closed affinity gap of RgIA4 across the rodent and human α9α10 nAChR.

## 3. Discussion

Conotoxin is a kind of peptide isolated from the venoms of marine cone snails that display potency and inherent selectivity at mammalian nAChRs. Based on their structure, function, and respective receptor target, conotoxins were divided into different classes. α-conotoxin is a class of conotoxin, which usually act as competitive antagonists in potential mechanisms of pain. α9α10 nAChR was implicated in pain and proposed to be a valid molecular target for pain-related drug development [24]. Early publications suggested that Vc1.1 and RgIA, two α-conotoxins, were reported to display the potential pain-relieving actions in a rat model [25,26]. Subsequently, in silico studies and electrophysiological experiments have further elucidated the binding properties of Vc1.1 and RgIA at the α9α10 nAChR [25,27].

Over the past decade, AChBPs, which showed a structural and functional homologue of ligand-binding domain of nAChRs, have been widely used as models for nAChRs [22]. Previously, there were many crystal structures of different α-conotoxins complexed with *Ac*-AChBP that revealed the key residues in α-conotoxins or nAChRs, namely for the GIC/*Ac*-AChBP complex [28], LvIA/*Ac*-AChBP complex [29], ImI/*Ac*-AChBP complex [30], and PnIA variant-*Ac*-AChBP complex [31]. Moreover, there are also many conotoxin analogs that were designed based on the AChBP-conotoxin structures. These include [S9A]TxID and [S9K]TxID for α3β4 nAChR [32,33], [S4A,E11A,L15A]MII for α6-containing nAChRs [34], and [S4K,N9A]Vc1.1 for α9α10 nAChR [35]. According to the considerable homology between AChBP and nAChR, especially in their ligand-binding domain, exploring the interactions between α-conotoxin and α9α10 nAChR can be less difficult. Although Marios Zouridakis et al. reported the X-ray structure of the extracellular domain of α9 subunit in complex with RgIA, they only showed the principal side in the RgIA/α9α10 nAChR interface. Here, we present two crystal structures of *Ac*-AChBP in complex with RgIA and RgIA4, respectively. It is the first time a complex between *Ac*-AChBP and pain-related conotoxin was reported and the first time the complementary side in RgIA/RgIA4-α9α10 nAChR interface through the homologous protein AChBP was shown.

Comparing RgIA/RgIA4-*Ac*-AChBP complexes with our previously reported conotoxin-AChBP complexes, the conotoxin bound *Ac*-AChBP showed similar conformation in its interface, even in loop C of *Ac*-AChBP (Figure S4A). All conotoxins between two adjacent AChBP subunits and its N-termini and C-termini were located at the bottom and top of the binding interface, respectively (Figure 2A,B). Among these α-conotoxins, the C-termini were superposed worse than those of N-termini. Through sequence alignment of GIC, LvIA, RgIA, and RgIA4, it is clearly shown that all conotoxin exhibited high homology in their N-termini (Figure S4B). However, the sequences in C-termini were significantly different. Thus, we think the sequence variations in C-termini may contribute to the unique selectivity of different nAChRs or determine its preferential receptor antagonism of α-conotoxin.

RgIA was firstly reported as a potent and specific blocker of the α9α10 nAChR [26]. Then, Romero HK et al. published an analog of RgIA, RgIA4, which displayed higher potency than RgIA for both human and rat α9α10 nAChR [26]. In this study, we determined the crystal structure of the complex of *Ac*-AChBP with RgIA and RgIA4, respectively, revealing the reason of the higher affinity and selective target of RgIA4. As shown in Figure 3, the local conformation of conotoxin changed a little when Arg9 and Tyr10 of RgIA were replaced with CIR9 and IYR10 of RgIA4, respectively. Whether in RgIA or RgIA4, it was obviously shown that Asp5, Arg7, and Arg11 play key roles in principal side. In the RgIA/*Ac*-AChBP complex, Arg9 and Tyr10 of RgIA interacted with *Ac*-AChBP mainly in complementary side. The marked shift was observed in position9 and position 10 of conotoxins. In the complementary side of RgIA/*Ac*-AChBP interface, Arg9 of RgIA interacted with Gln55 and Met114 of *Ac*-AChBP through Van der Waal contact. When CIR9 was introduced into the conotoxin backbone, it formed hydrogen bonds with Thr34 and Gln55, respectively (Figure 3B,D and Table 1). Similar to Arg9 in RgIA, substitute Tyr10 of RgIA to IYR introduced additional interaction between IYR10 of RgIA4 and Arg77 of *Ac*-AChBP. Thus, the side chains change of Arg9 and Tyr10 of RgIA may compensate for the affinity increase of RgIA with the receptor, which explains the observation that RgIA4 produced a complete block of the nAChR with low nanomolar potencies [15].

Notably, the ligand-binding domains of α9 and α10 subunits had a remarkable sequence similarity with *Ac*-AChBP (Figure 2D). Given that RgIA and RgIA4 are specific antagonists to α9α10 nAChR, we performed MD simulations based on crystal structures of RgIA/*Ac*-AChBP and RgIA4/*Ac*-AChBP, respectively, to assess the possibility of conotoxins for any of these sites. Previously, RgIA was reported to show a favorable binding at α9(+)/α9(−) or α10(+)/α9(−) rather than the traditional α9(+)/α10(−) interface based on the electrostatic potential distribution analysis [17]. Because RgIA4 is the analog of RgIA, the electrostatic distribution of RgIA4 may be similar to RgIA. Together with the previous research and our contact analysis, the α9(+)/α10(−) interface in RgIA4-α9α10 nAChR interaction is also undesired. Combined with sequence alignment and MD simulation, key residues of *Ac*-AChBP, α9 and α10 subunits in the principal side were highly conserved in nAChR subunits and *Ac*-AChBP, such as Tyr95, Trp151, Tyr192, Glu197, Tyr199, and Pro200 of α9 or α10 subunits. It is worth mentioning that the importance of the abovementioned conserved residues were mostly evaluated by mutational studies previously. Mutated Trp151 to threonine in the α9 subunit and their co-expression in oocytes was less potently inhibited by RgIA [13]. Additionally, the single mutation of α10-Glu197 or α10-Pro200 to glutarnine led to 25- and 300-fold decreased sensitivity to RgIA [36]. Additionally, when α10 subunits act as (+) side in RgIA/α9α10 nAChR complex, the important interaction between Arg7 of RgIA and Asp201 of α10 subunit cannot be ignored, though there are no functional analyses of Asp201. In contrast to principal side, most interacted residues in the complementary side of the RgIA/RgIA4-α9α10 nAChR interface are significantly different with nAChR subunits or *Ac*-AChBP. The main interaction on this side are hydrogen bonds and the interacted residues involved here are poorly investigated. On this side, only Asp121 of the α9 subunit is previously demonstrated to have critical roles through mutation analyses [36]. Apart from Asp121, Thr38, Trp57, Arg58, Arg81, Asp168, Ser170, and Asp171 of α9 subunit also show their importance in binding RgIA/RgIA4 in our MD simulation in RgIA-α9α10 nAChR complex and RgIA4-α9α10 nAChR complex. Among these residues, Trp57, Arg81, Asp168 and Asp171 are highly conserved in most nAChR subunits, and S170 is unique in the α9 subunit. Compared with the model of the RgIA-α9α10 nAChR complex with RgIA4/α9α10 nAChR complex, Ser79 and Arg113 of the α9 subunit are specifically exist in the model RgIA4/α9α10 nAChR complex. Interestingly, position 79 and 113 in the β2 and β4 subunits are previously reported to affect the sensitivity of receptor to α-conotoxin RegIIA [37,38]. Thus, Ser79 and Arg113 of the α9 subunit may also be important in increasing the affinity to conotoxins.

Taken together, this is the first time the structure of the antagonist of α9α10 nAChR with *Ac*-AChBP was shown. The structures show the key residues of RgIA/RgIA4 in binding *Ac*-AChBP. Based on the structures of the complex, we performed MD simulation and revealed the interactions between RgIA/RgIA4 and α9α10 nAChR in detail and proposed the important residues in the complementary side. These results may be valuable in the design and development of potent α9α10-selective drugs, with significant implications for the treatment of neuropathic pain.

## 4. Methods and Materials

### 4.1. Overall Structure of RgIA and RgIA4 Bound to Ac-AChBP

Linear peptides were assembled using solid-phase methodology on an ABI 433A peptide synthesizer (Applied Biosystems Inc., Foster City, CA, USA). Cys residues were protected in pairs with S-trityl (Trt) on CysI and CysIII (the first and third Cys) and S-acetamidomethyl (Acm) on the second and fourth Cys. The first disulfide bond (CysI-CysIII) was formed under an oxidative condition using 20 mM potassium ferricyanide K_3_[Fe(CN)_6_], and 0.1 M Tris-HCl, pH 7.5, which was reacted for 45 min, and the monocyclic peptide was purified by reverse-phase HPLC. Then, the Acm groups were cleaved and the second disulfide bond (CysII-CysIV) was formed by iodine oxidation. The bicyclic peptide was purified by HPLC on a reversed-phase C18 Vydac column (Agilent Technologies, Hesperia, CA, USA) with a linear gradient of 10–40% B90 in 30 min. Solvent A was 0.1% TFA in H_2_O, Solvent B include 90% CAN, and 0.092% TFA in H_2_O. The purity of final product was confirmed by reversed phase-HPLC (Waters ACQUITY UPLC H-Class) and ESI-IT-TOF (Shimadzu, Tokyo, Japan) mass spectrometry.

### 4.2. Protein Expression and Purification

The *Ac*-AChBP was expressed using the Bac-to-Bac baculovirus system (Invitrogen). *Ac*-AChBP with an N-terminal gp67 signal peptide was used to facilitate secretion and a C-terminal six-histidine tag was cloned into the pFastBac Dual vector (Invitrogen) using ClonExpress II One Step Cloning Kit (Vazyme Biotech Co., Ltd., Nanjing, China). The construct was transformed into bacterial DH10Bac competent cells, and the extracted bacmid was then transfected into Sf9 cells in the presence of Cellfectin II Reagent (Invitrogen) to produce recombinant baculoviruses. After two cycles of amplification, the high-titer viruses were used to infect Hi5 insect cells with the density of 2 × 10^6^ cells per ml in HyQ SFX medium (HyClone). The supernatant of cell culture containing *Ac*-AChBP was harvested 48 h after infection and purified with nickel-nitrilotriacetic acid resin (GE Healthcare, Chicago, IL, USA) in HBS buffer (10 mM Hepes, pH 7.2, 150 mM NaCl), followed by gel-filtration chromatography using the Superdex 200 column (GE Healthcare). Purified *Ac*-AChBP and synthetic conotoxins were mixed with a molar ratio of protein to conotoxin equal to 1:2.5, then left on ice overnight. Gel-filtration chromatography was utilized again to obtain the conotoxin/*Ac*-AChBP complex.

### 4.3. Crystallization, Data Collection and Structural Determination

The purified α-CTx/*Ac*-AChBP complex was concentrated to ~20 mg/mL for crystallization. The crystals were grown at 18 °C by mixing an equal volume of protein and reservoir solutions using the sitting drop vapor diffusion method. For crystal growth of the RgIA/*Ac*-AChBP complex, the reservoir solution contained 1.8 M magnesium sulfate hydrate and 0.1 M sodium acetate trihydrate pH 4.6. For the RgIA4/*Ac*-AChBP complex, the reservoir solution contained 1.8 M sodium phosphate monobasic monohydrate and potassium phosphate dibasic, pH5.0. All crystals were cryoprotected in well solution supplemented with 20% (vol/vol) glycol and were cooled to liquid-nitrogen before data collection. All diffraction data were collected on the BL17U1 beamline at Shanghai Synchrotron Research Facility (SSRF) [39] and processed with HKL2000 [40]. The structures of α-CTx/*Ac*-AChBP complexes were determined by the molecular replacement method with the crystallographic program PHASER in the CCP4 suite [41]. The program PHENIX and COOT were used to iterative refinement of all structures [42,43]. Structural validations were performed with the program MolProbity [44]. All structural figures used here were generated with PYMOL (http://www.pymol.org/, accessed on 4 November 2021). All diffraction data collection, processing, and structural refinement statistics are listed in Table S1.

### 4.4. Homology Modeling and Model Refinement

The homology models of the extracellular ligand-binding domain of human α9α10 nAChR bound to RgIA4 and RgIA were generated using the Swiss-Model. The crystal structures of *Ac*-AChBP in complex with RgIA4/RgIA were used as templates to model the the RgIA/RgIA4-bound α9α10 nAChR. Subsequently, generated models were checked by PDBsum, which includes a full PROCHECK assessment of each protein’s geometry. For model refinement, force field ff14SB was used. For noncanonical residues, force field library and parameters were generated by R.E.D. Server Development [45,46]. In this pipeline, partial charges are calculated by constructing ACE/NME (ACE = CH3CO and NME = NHCH3) capped amino acid (the central fragment is new amino acid). By adding two intra-molecular charge constraints (equal 0), we could acquire central fragment atom charges and then the force field library. When setting up molecular dynamic simulation, we used the GPU-accelerated software AMBER v18 and introduced the ff14SB force field and the new force field together [47,48]. The complexes were solvated in the SPCE water box. Sodium ions were added to neutralize the systems. The systems were minimized with 1000 steps twice and the first minimization was performed with the solute restrained to their position by a harmonic force of 500 kcal/mol∙Å^2^. The second minimization was then performed with all position restraints withdrawn. The systems were then gradually heated up from 0 to 1000 K over 40 ps with restrained to the position of potential non-interaction residues using a 500 kcal/mol∙Å^2^ force potential and then gradually cooled down to 0 K over 120 ps with the same restraint. The MD-simulation of these two processes used a time step of 1 fs. Another minimization was conducted without position restraint through 500 steps.

## Figures and Tables

**Table 1:** Contacts between residues of *Ac*-AChBP and RgIA and RgIA4, respectively.

*Ac*-AChBP	RgIA	RgIA4
Principal Side		
Tyr91	Arg7	Arg7
Trp145	Pro6, Arg7	Pro6
Val146	Arg7	
Gln184	Asp5, Arg7	
Tyr186	Asp5	Gly1, Asp5
Cys188		Cys2
Cys189	Cys8, Arg11	Cys2, Cys8
Glu191	Tyr10, Arg11	Arg11
Tyr193	Cys8	Cys8, Arg11
Ile194	Arg7	
Complementary Side		
Thr34		CIR9
Gln55	Arg9	CIR9, Arg13
Arg57	Arg13	Arg13
Asp75	Tyr10	IYR10
Arg77		IYR10
Met114	Arg9	
Asp162	Ser4	Ser4
Ser164	Ser4	
Ser165	Ser4	Ser4

## Supplementary Materials

The following is available online at https://www.mdpi.com/article/10.3390/md19120709/s1. Figure S1: The HPLC and mass spectra profiles of RgIA/RgIA4; Figure S2: Ramachandran plot of the homology models of two possible stoichiometries (α9)_2_(α10)_3_ and (α9)_3_(α10)_2_; Figure S3: The molecular dynamics models of human α9α10 nAChR bound to RgIA; Figure S4: Comparison of different α-CTxs bound by *Ac*-AChBP; Table S1: The relevant data collection and refinement statistics of two X-ray crystal structures.
