# Supplementary material for: From Crystal Structures of RgIA4 in Complex with Ac-AChBP to Molecular Determinants of Its High Potency of α9α10 nAChR
Source: Mar Drugs. 2021 Dec 17;19(12):709. doi: 10.3390/md19120709 (PMC8709174; doi:10.3390/md19120709)
Supplement: Supplementary file 1 [file marinedrugs-19-00709-s001.zip › marinedrugs-1473603-supplementary.pdf]

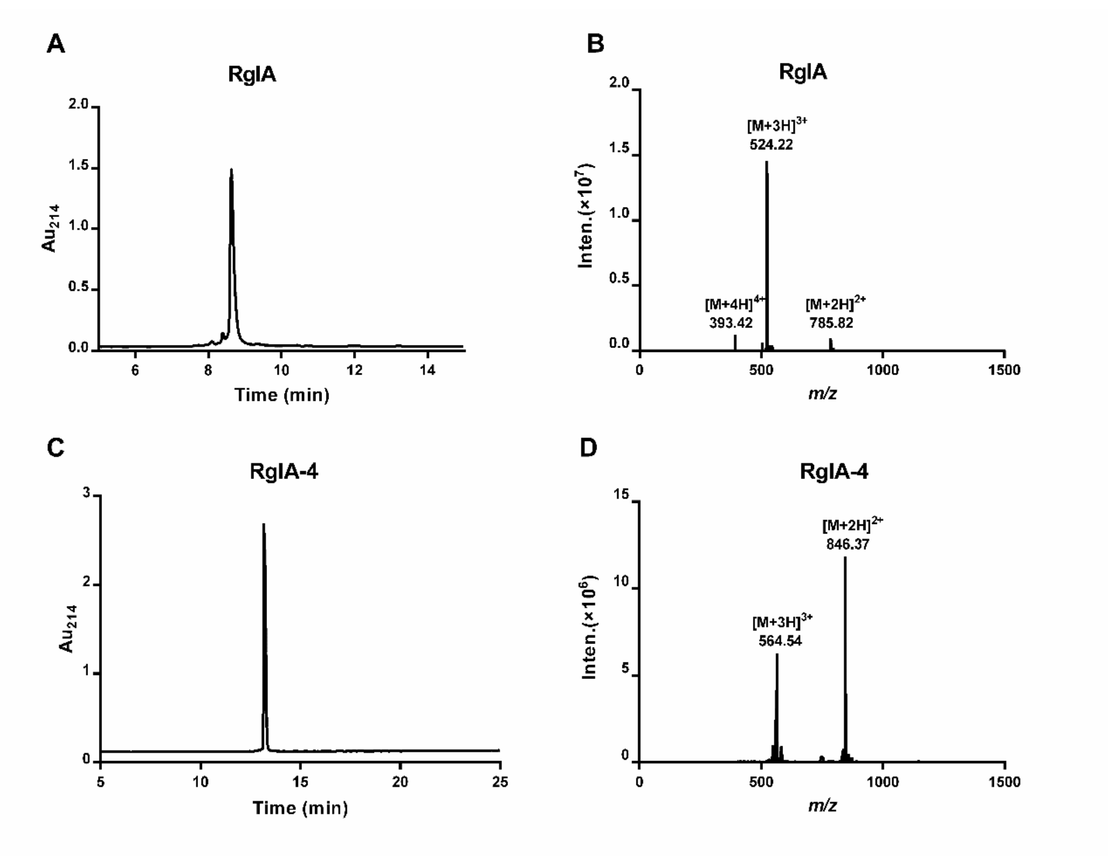

Figure S1. HPLC and mass spectra profiles of RgIA/RgIA4. A, C HPLC chromatograms of RgIA and RgIA4, respectively. **B, D** Electrospray ionization mass spectrometry (ESI-MS) data for RgIA and RgIA4, respectively.

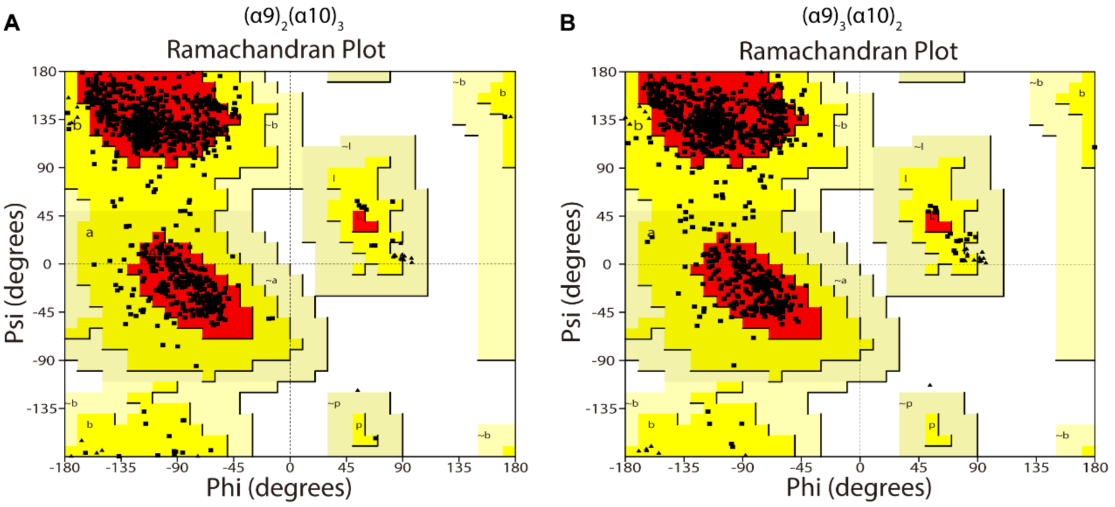

Figure S2. Ramachandran plot of the homology models of two possible stoichiometries $(\alpha9)_2(\alpha10)_3$ (A) and $(\alpha9)_3(\alpha10)_2$ (B). Red: Residues in most favored region; Dark yellow: Residues in additional allowed regions; Pale yellow: Residues in generously allowed regions.

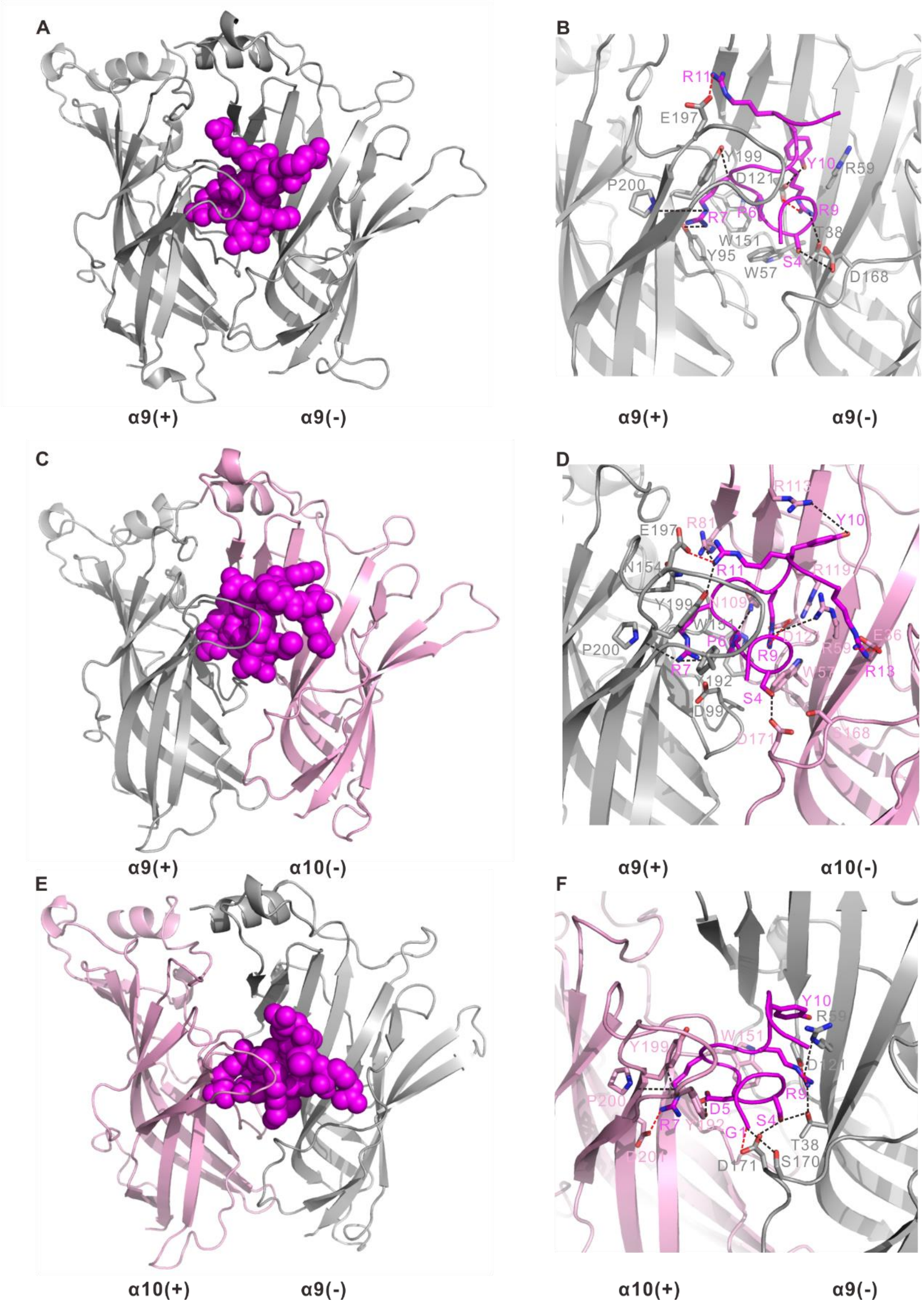

Figure S3. Molecular dynamics models of human α9α10 nAChR bound to RgIA. A, B RgIA bound to the α9(+)/α9(-) interface. **C, D** RgIA bound to the α9(+)/α10(-) interface. **E, F** RgIA bound to the α10(+)/α9(-) interface. hydrogen bonds and salt bridges were represented by black dashed lines and red dashed lines, respectively.

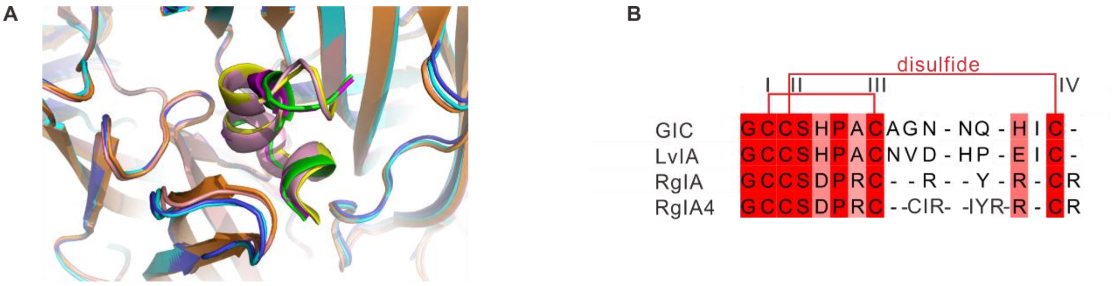

Figure S4. Comparison of different α-CTxs bound by *Ac*-AChBP. A Comparison of Ac-AChBP with four conotoxins. RgIA is showed in magenta, RgIA4 in green, GIC in pink, and LvIA in yellow. **B** Multiple sequence alignment of RgIA, RgIA4, GIC and LvIA. Disulfide bridges are showed in Cys1-Cys3 and Cys2-Cys4.

Table S1. Data collection and refinement statistics.

	**RgIA-*Ac*-AChBP**	**RgIA4-*Ac*-AChBP**
**Data collection**		
Beamline	SSRF BL17U	SSRF BL17U
Wavelength	0.9796 Å	0.9796 Å
Space group	C 1 2 1	C 1 2 1
Cell dimensions		
a, b, c (Å)	206.271, 135.955, 132.067	206.149, 136.529, 131.111
α, β, γ (°)	90, 101.162, 90	90, 100.566, 90
Resolution (Å)	50.00-2.50	50.00-2.60
[a]R_{merge}	0.110(0.792)	0.204(1.418)
[b]R_{pim}	0.063(0.466)	0.083(0.580)
[c]$CC_{1/2}$ of the highest resolution shell	0.992(0.672)	0.987(0.648)
I/δI	10.980(1.804)	11.694(2.290)
Completeness (%)	98.36 (99.83)	98.20 (93.27)
Redundancy	3.9	7.0
**Refinement**		
Resolution (Å)	35.4 - 2.503 (2.592-2.503)	48.26 – 2.611 (2.704 - 2.611)
No. reflections	121025 (11626)	106333 (10042)
R-work	0.1727 (0.2565)	0.1840 (0.2475)
R-free	0.2214 (0.3164)	0.2251 (0.3073)
No. atoms	17680	17319
macromolecules	17164	16872
ligands	10	76
solvent	506	371
Wilson B-factor (Å^2)	35.34	36.94
Average B-factors (Å^2)	41.74	39.48
macromolecules	41.79	39.36
ligands	42.97	70.64
solvent	39.78	38.42
r.m.s. deviations		
Bond lengths (Å)	0.008	0.010
Bond angles (°)	1.03	1.38
Ramachandran plot (%)		
Favored	96.42	95.80
Allowed	3.30	3.67
Outlier	0.28	0.53
